# *Centella asiatica* phenolic extract-mediated bio-fabrication of silver nanoparticles: characterization, reduction of industrially relevant dyes in water and antimicrobial activities against foodborne pathogens

**DOI:** 10.1039/c9ra08618h

**Published:** 2019-11-20

**Authors:** Fredrick Nwude Eze, Adesola Julius Tola, Ozioma Forstinus Nwabor, Titilope John Jayeoye

**Affiliations:** Department of Chemical Sciences, Joseph Ayo Babalola University Ikeji-Arakeji Osun State Nigeria; Department of Chemistry, Biochemistry and Physics, Université du Québec à Trois-Rivières (UQTR) Trois-Rivières Québec G9A 5H7 Canada; Department of Microbiology, University of Nigeria Nsukka Nigeria nwaborozed@gmail.com; Department of Chemistry/Biochemistry/Molecular Biology, Alex Ekwueme Federal University, Ndufu Alike-Ikwo Abakaliki Ebonyi State Nigeria titioye040@yahoo.com

## Abstract

In this article, we have reported an environmentally benign and cost-effective method for the synthesis of monodispersed silver nanoparticles (AgNPs), based on *Centella asiatica* phenolic extracts (CAPE). The presence of phenolics was confirmed by ultra high-performance liquid chromatography coupled with electrospray ionization quadrupole time of flight mass spectrometry (UHPLC-ESI-qTOF-MS). Colloidal AgNPs synthesized under different concentrations of silver nitrate were monitored with a UV-vis spectrophotometer. Maximum absorption spectra intensity was found to range between 430–440 nm, during a synthesis time of 90 minutes at room temperature. The as-synthesized CAPE-AgNPs, was subjected to various instrumental characterizations such as, transmission electron microscopy (TEM), X-ray powder diffraction (XRD), energy dispersive X-ray spectroscopy (EDS), Fourier transform infrared (FTIR) spectroscopy, dynamic light scattering (DLS) and zeta potential. At the optimized synthesis conditions, spherical and monodispersed CAPE-AgNPs were obtained, with an absorption maximum at 430 nm. The crystalline CAPE-AgNPs had a face-centered-cubic (fcc) crystallographic structure, possessing average sizes estimated from TEM, to be between 20–25 nm diameter, a hydrodynamic diameter from DLS of about 90 nm and a zeta potential value of −28.7 mV. FTIR results validated the presence of phenolics on the surfaces of CAPE-AgNPs. The anti-microbial capacity of CAPE-AgNPs was further demonstrated on different pathogenic bacterial strains with satisfactory performances. As a result of the high surface area to volume ratio of CAPE-AgNPs, it was investigated as a catalyst towards the reduction of prominent environmental pollutants, 4 nitrophenol (4 NP), Congo red (CR) and methylene blue (MB). Pseudo first order kinetics were obtained with rate constants of 3.9 × 10^−3^ s^−1^ for 4 NP, 54.7 × 10^−3^ min^−1^ for MB and 5.6 × 10^−3^ s^−1^ for CR. The catalytic performance and antimicrobial activities of CAPE-AgNPs suggest its potential application in wastewater treatment and control of pathogenic microbes.

## Abstract 2

Illustration of biogenic synthesis of AgNPs based on *Centella asiatica* phenolic extract and applications.

## Graphical abstract

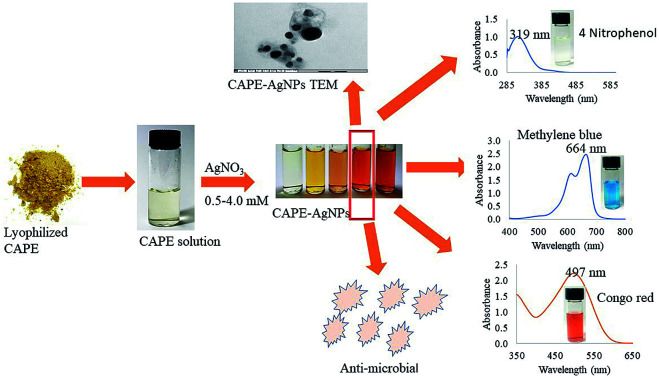

## 1. Introduction

Environmental pollution is a major global challenge facing humanity today which results from man's indiscriminate use of resources. Azo dyes and nitrophenols arising from textile, paper, cosmetics, ceramics or explosives manufacturing and industrial processes constitute a substantial part of these worrisome pollutants.^1–3^ It has been estimated that about a hundred thousand types of dyes are produced yearly corresponding to an aggregated 700 000 to 1 000 000 million tons for applications in rubber, plastic, paint, pigment, leather and paper industries.^4^ Moreover, it is estimated that between 10–15% of the dyes produced are released into water bodies constituting various forms of carcinogens, mutagens and bio-refractive pollutants that might cause deleterious health consequences to humans and aquatic life forms.^1–3,5,6^ Thus, rendering these compounds less hazardous prior to disposal or during wastewater treatment is critical for safeguarding human and environmental wellbeing. Traditionally, remediation of dye effluents had generally involved chemical and physical means such as, adsorption, ozonation, coagulation, reverse osmosis or precipitation. However, owing to the poor aqueous solubility and high resistance to degradation of these organic compounds,^6–8^ efficiently decolorizing and transforming them into innocuous forms using these conventional approaches have been limited by high cost, production of toxic by-products, and strict energy requirements.^9–11^ It is against this backdrop that research into eco-friendly, safe, more cost-effective and efficient, alternative approaches remain highly relevant. The use of metal nanomaterials in the presence NaBH_4_ for the chemical reduction and catalytic degradation of dye pollutants is currently gaining traction as a clean and an efficient approach.^12,13^

The development of environmentally benign nanomaterial with multi-applications owing to their unique chemical, physical, optical or thermodynamic characteristics is gaining prominence is diverse fields in modern research spanning catalysis,^14^ targeted drug delivery,^15^ diagnostics,^16^ sensing^17^ and antimicrobials. Given their high surface to volume ratios, noble metal nanoparticles can enhance the reactions between reactants as catalysts. As a result, efforts are continuously devoted to improving synthesis cost, by exploiting different reducing and capping agents. The conventional synthesis methods of metal nanoparticles are limited in terms of energy demand, cost of reducing agents used; which are oftentimes toxic to the environment and disruption in the surface architecture of the synthesized nanoparticles.^18,19^ Over the last few decades, metal nanoparticles synthesis from biomaterials have been popularized as alternative to the physical and chemical approaches with silver nanoparticles receiving the most attention.^20,21^ The abundance of biomolecules with varied natural constituents from renewable resources lend credence to the present zest in researches on green nano-synthesis. To this end, plethora of biomaterials especially from plants have been investigated for synthesis of biomolecule-stabilized and capped AgNPs.^22–24^

*C. asiatica* is a small perennial herbaceous medicinal plant belonging to the Umbelliferae family commonly found in tropical marshes across Africa and Asia. Over the millennia it has served both medicinal and nutritive functions. *C. asiatica* is endowed with vast array of bioactive metabolites including phenolics such as gallic acid, quinic acid, chlorogenic acids, catechin, quercetin kaempferol. It is also rich in triterpenes *viz.*, asiatic acid, medecassic acid, asiaticoside, madecassoside.^25,30^ The antioxidative and reductive abilities of *C. asiatica* has been established in numerous studies, thus suggesting its potential for reductive biosynthesis.

Owing to the abundance of the aforementioned phytoconstituents in *C. asiatica* putatively capable reductive biosynthesis with better safety profile, we thus seek to report objective of the current study was to describe the bio-fabrication of silver nanoparticles from *C. asiatica* phenolic extract (CAPE) without any costly, hazardous or toxic stabilizing or capping agents. In comparison with other numerous works on green synthesis of metals nanoparticles, the sample extraction adopted in this work is completely green. This phenolics extraction step distinguished this work from others. As a result, only a small quantity of the bio-reductant was used in the synthesis proper. Finally, the CAPE mediated biogenic silver nanoparticles (CAPE-AgNPs) was characterized and investigated as a heterogenous catalyst for the reduction of organic dyes (4-nitrophenol, Congo red and methylene blue) in water at room temperature and its antimicrobial activities against pathogenic foodborne bacteria. The results from this work provide insights into the benign and eco-friendly synthesis CAPE-AgNPs and its potential implications in the control of environment pollution and pathogenic foodborne microbes.

## 2. Experimental

### 2.1. Plant materials and preparation of *C. asiatica* phenolic extract (CAPE)

Fresh *C. asiatica* aerial parts was obtained locally and processed as previously reported.^26^ The plant sample was washed with tap water and rinsed with reverse osmosis water. The aerial parts were air-dried to reduce moisture content for 12 hour and further oven dried at 60 °C for another 12 hours. The dried sample was ground into fine powder and stored in an opaque bottle at −20 °C for further extraction.

**Fig. 1:**
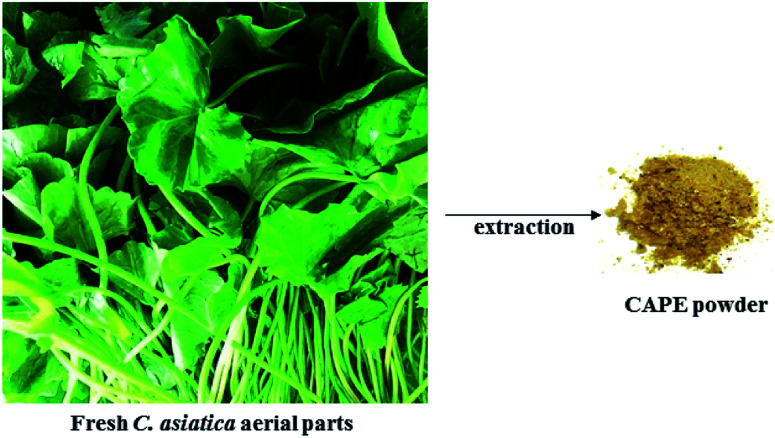
Preparation of CAPE from *C. asiatica*.

Dried *C. asiatica* powder (50 g) was extracted using 500 mL of ethanol/water mixture (80 : 20 v/v) for 2 hours while gently stirring with an overhead stirrer. The mixture was filtered using a Buchner funnel overlaid with Whatman No 1 filter paper. The collected marc was re-extracted. The combined filtrate was concentrated using rotary evaporator at 40 °C to one-third of its original volume. Thereafter, the filtrate was kept at 4 °C overnight. A clear supernatant was obtained and subjected to reduced pressure in a speed-vac in order to remove any residual organic solvent. The mixture was filtered using Whatman No. 1 filter paper under gravity and then lyophilized to give a light brown powder (Fig. 1) hereafter referred to as *C. asiatica* phenolic extract (CAPE). CAPE was stored at −20 °C protected from light.

### 2.2. Chemical characterization

#### 2.2.1. Determination of total phenolic and flavonoid contents

CAPE was analysed for its total phenolic and flavonoids contents *via* Folin-Ciocalteu and aluminium chloride colorimetric assays, respectively. To determine the phenolic content, CAPE (10 μg μL^−1^) and the standard-gallic acid (1 μg μL^−1^) were prepared in methanol/water (50 : 50 v/v). Briefly, 100 μL of sample, blank or standard was added to the test tubes. Thereafter, 200 μL of Folin-Ciocalteu reagent (10% v/v) was added and vortexed. After 5 min of incubation, the mixture was supplemented with 800 μL of sodium carbonate, vortexed and incubated in the dark at room temperature for 2 hours. 200 μL of the blue mixture formed was pipetted into a 96-well microplate and the absorbance was read at 765 nm using microplate reader. A standard curve for amount gallic acid (μg) *versus* absorbance at 765 nm was generated and used to determination of the total phenolic content of the sample.

To determine the flavonoid content, CAPE (10 μg μL^−1^) and the standard – quercetin (1 μg μL^−1^) were prepared in 100% methanol. The CAPE or quercetin solution (30 μL) was diluted with 160 μL of methanol and subsequently, 10% aluminium chloride (30 μL), 1 M sodium acetate (30 μL), and distilled water (850 μL) were added. The mixture was vortexed and incubated in the dark for 30 min before reading the absorbance at 415 nm in a UV-visible spectrophotometer. Absorbance reading *versus* concentration of standard was used to obtain a calibration curve from which the total flavonoid content was derived.

#### 2.2.2. DPPH free radical scavenging activity (DPPH assay)

CAPE was investigated for its ability to scavenge free radicals using 2,2-diphenyl-1-picrylhydrazyl (DPPH) assay as previously described.^27^ In brief, 100 μL of DPPH solution (0.1 mM) in methanol was added into 100 μL serial dilutions (10–2000 μg mL^−1^) of CAPE in a 96-well plate. Thereafter, a microplate reader was used to measure the sample absorbance at 517 nm after incubation at 25 °C for 30 min. The result was reported as IC_50_, that is the CAPE concentration which scavenges fifty percent of the radical.

#### 2.2.3. Ferric reducing radical antioxidant power (FRAP)

The antioxidant ability of CAPE determined by its capacity to reduce ferric (Fe^3+^)-TPTZ complex to a ferrous (Fe^2+^) form using FRAP assay as described by Benzie and Strain.^28^ CAPE solution prepared in 50% methanol or the standard – Trolox (up to 600 μmol L^−1^) prepare in distilled water were added (10 μL) into FRAP solution (200 μL) and incubated at 37 °C in the dark. The absorbance was read at 593 nm and CAPE antioxidant potential was expressed as μmol of Trolox equivalent per gram of CAPE.

#### 2.2.4. Ultra-high-performance liquid chromatography coupled to diode array detection and electrospray time-of-flight tandem mass spectrometry (UHPLC-DAD-ESI-QTOF-MS) analysis

CAPE (40 mg mL^−1^) was prepared in 50% aqueous methanol. The solution was centrifuged at 10 000 rpm for 8 minutes. The supernatant was filtered through a 0.22 μM nylon filter to obtain a clear solution which was used for LC-MS analysis.

The UHPLC-ESI-qTOF-MS analysis of CAPE was performed as earlier reported^34^ with slight modifications. Separation of chemical components from CAPE was carried out on an Agilent 1290 Infinity II LC System (Agilent Technologies, Santa Clara, CA) equipped with an autosampler, vacuum degasser, binary pump, thermostat and diode array detector. The sample was resolved using Agilent's ZORBAX Eclipse Plus C18, 150 × 2.1 mm, 1.8 μm column with acidified Milli-Q water (0.1% formic acid) and acetonitrile as mobile phases A and B, respectively. The elution gradient was set up as follows: 0.50 min, 0.00% B; 16.50 min, 100.00% B; 17.50 min, 100.00% B; 20.00 min, 0.00% B; 22.00 min, 0.00% B. The injection volume was 2.0 μL, the flow rate and temperature were maintained throughout the separation at 0.20 μL min^−1^ and 25 °C, respectively.

The HPLC system was coupled to an Agilent 6545 LC/Q-TOF MS mass spectrometer equipped with a Dual Agilent Jet Stream ESI operating in negative mode, covering spectra with mass range from *m*/*z* 100 to 1500 at a scan rate of 1.00 spectra per s. Accurate mass measurements by the instrument was ensured using an automated calibrant delivery system that continuously introduced a reference solution with mass mix of *m*/*z* 112.98558700 (TFA anion) to *m*/*z* 1033.98810900 (HP-0921) in the negative ESI mode, while *m*/*z* 121.05087300 (purine) and *m*/*z* 922.00979800 (HP-0921) were introduced in positive ESI mode. The ESI-MS parameters were as follows: drying gas temperature, 325 °C; drying gas flow rate, 13 L min^−1^; nebulizer gas pressure 35 psig; capillary voltage, 4000 V; fragmentor voltage, 175 V; radiofrequency voltage in the octupole (OctopoleRFPeak), 750 V and fixed collision energies of 10.00 eV, 20.00 eV and 40.00 eV. Data acquisition done using Mass Hunter Workstation Software Data Acquisition for Q-TOF version B.08.00 (B8058.3 SP1) and QTOF Firmware version 20.712.

### 2.3. Synthesis of silver nanoparticles

Biogenic silver nanoparticles were synthesized using CAPE as the reductant and stabilizer following a previous work^29^ with minor modification. Briefly, a 5.0 mg mL^−1^ of lyophilized CAPE sample was dissolved in ethanol : water mixture (1 : 3). Then different concentrations of AgNO_3_ were prepared (0.5–4.0) mM. 95 mL of AgNO_3_ was measured into a 250 mL beaker wrapped with an aluminium foil to prevent photo-oxidation and placed on a magnetic stirrer. CAPE solution (5.0 mL) adjusted to a pH 9.0 was added to the AgNO_3_ solution and stirred at room temperature for 90 minutes. Successful synthesis of CAPE-AgNPs was monitored with a UV-visible spectrophotometer. Parameters affecting CAPE-AgNPs, such as the effect of AgNO_3_ concentration and synthesis kinetics were briefly optimized. CAPE-AgNPs was centrifuged at 15 000 rpm for 15 minutes and was washed with an ultra-pure water to remove excess CAPE and free AgNO_3_ that were not used for AgNPs synthesis from solution. The collected pellets were lyophilized prior to characterization and applications.

### 2.4. Materials characterization

CAPE-AgNPs was subjected to various instrumental characterizations to obtain vital, detailed information such as the shape and morphology of the synthesized nanoparticles. Transmission electron microscopy (TEM), images were observed on a JEM-2010 electron microscope from JOEL Ltd., Japan. Samples were prepared by spotting about 5 μL of colloidal solution of CAPE-AgNPs on a copper grid, then evaporating solvent at ambient temperature. UV-vis absorption spectra were obtained from a UV-2600 spectrophotometer (Shimadzu, Japan), with a 1.0 cm path length quartz cell. Particles hydrodynamic diameters and zeta potential were recorded with a Brookhaven's NanoBrooks ZetaPALS (Brookhaven instrument, USA). Colloidal solution of CAPE-AgNPs in cuvette was inserted into the sample holder of the equipment and the particle size was measured with a size measuring software, while the zeta potential was obtained using the zetasizer software.

X-ray diffraction (XRD) of CAPE-AgNPs pellets was performed on an Empyrean XRD diffractometer (Empyrean, Netherland), for 2 theta value ranging from 20–80° using Cu Kα with a radiation wavelength of 0.154 nm and a scan speed of 70.2 seconds. Qualitative elemental compositions of CAPE-AgNPs were obtained using an energy dispersive X-ray spectroscopy (EDS) on Oxford instruments. Fourier transform infrared (FTIR) spectra were collected using a PerkinElmer spectrum BX, in the range of 400–4000 cm^−1^, with 4.0 cm^−1^ resolution. Powdered CAPE-AgNPs was ground with potassium bromide (KBr) into a thin circular film and was placed in the sample holder of the equipment.

### 2.5. Biological application (antimicrobial activity)

#### 2.5.1. Antimicrobial activities of CAPE and CAPE-AgNPs

The antimicrobial activities of CAPE and the CAPE-AgNPs were determined using the standard broth micro-dilution method.^30^ Foodborne pathogenic bacteria including, *Listeria monocytogenes* F2365, *Escherichia coli* O157:H7, *Staphylococcus aureus* ATCC 25923 and an isolate of *Bacillus cereus* and *Pseudomonas aeruginosa* obtained from the Natural Products Research Centre of Excellence, Faculty of Science, Prince of Songkla University were used in the assay. The test bacteria were cultured in Tryptic Soy Broth (TSB) to log growth phase and adjusted to 10^6^ CFU mL^−1^ in MHB. An aliquot (100 μL) of bacterial suspension was seeded into individual wells containing serially diluted CAPE and AgNPs in a 96-well micro-titer plates and incubated for 18 h at 37 °C. The minimum inhibitory concentrations (MIC) values were recorded as the lowest concentration that completely inhibited the bacteria growth. The minimum bactericidal concentrations (MBC) were determined using the spot plate technique by seeding 10 μL aliquots from wells without growth on TSA. The plates were incubated at 37 °C for 18 h. The MBC values were recorded as the lowest concentrations that showed no growth on TSA plates. All experiments were set up in triplicates for two independent studies.

### 2.6. Catalytic applications

The catalytic efficacy of CAPE-AgNPs was demonstrated on notable environmental pollutants 4-nitrophenol (4-NP) and azo dyes (Congo red, CR and methylene blue, MB). Freshly prepared sodium borohydride (NaBH_4_) was used as the reducing agent. For the catalytic reduction of 4-NP, into a 3.0 mL glass vial, 1.4 mL of 215 μM of 4-NP was added, then 1.4 mL of 21.5 mM of NaBH_4_ was added, followed by the addition of 200 μL CAPE-AgNPs. The mixture was vortexed, then transferred into a cuvette and the UV-vis spectra recorded every 1 minute. For catalytic degradation of CR, 700 μL of 215 μM of CR, were mixed with 700 μL of 33.0 mM of NaBH_4_. 700 μL of ultra-pure water was added to the mixture, after which 200 μL of CAPE-AgNPs was added to complete 3.0 mL reaction mixture. The absorption spectra of the reaction mixture were read at 1 minute interval. For MB catalytic degradation, 700 μL of 215 μM of MB was added to 700 μL of 21.5 mM of NaBH_4_, then 1200 μL of ultra-pure water was added, followed by 400 μL of CAPE-AgNPs. The absorption spectra of the mixture were read at 2 minutes interval.

## 3. Results and discussion

### 3.1 Chemical characteristics of CAPE

Numerous studies have demonstrated the synthesis of silver nanoparticles from phytoconstituents. While the precise mechanism underlying the photosynthesis of silver nanoparticles remains to be fully elucidated, literature data suggest polyols, chiefly flavonoids and phenolic compounds are largely responsible for the reduction of silver ions.^31,32^ As shown in Table 1, CAPE is endowed with phenolics and flavonoids, which in part explains its antioxidant activity and reduction potential, as revealed by the DPPH and FRAP values, respectively. CAPE's ability to reduce the ferric-TPTZ complex foreshadowed its potential to reduce silver ions towards the biogenic synthesis of nanoparticles.

**Table 1:** Chemical characteristics of CAPE

Characteristics	CAPE
Yield (%) in dry weight	24.6
Phenolic content (mg gallic acid g^−1^)	22.13 ± 0.25
Flavonoid content (mg quercetin g^−1^)	9.43 ± 0.12
FRAP (μmol Trolox)	190 ± 8.39
DPPH [IC_50_] μg mL^−1^	310.08 ± 0.68

The UHPLC-ESI-qTOF-MS analysis revealed the identities of the individual biomolecules present in CAPE. As shown in Table 2, CAPE is mainly composed of phenolic compounds ranging from simple phenolics such as quinic acid, chlorogenic acids to complex polyphenols such as the flavonoid glycoside, quercetin 3′-*O*-glucuronide and condensed tannin, procyanidin B1. In addition, triterpenoids including, asiaticoside and madecassoside were part of CAPE. Previously it had been suggested that the most probable mechanism of a phyto complex such as CAPE mediated silver nanoparticle synthesis would involve the various chemical constituents working together in synergy and simultaneously serving as reducing and stabilizing agents.^33,34^

**Table 2:** Profile of CAPE chemical constituents based on UHPLC-ESI-QTOF-MS analysis^a^

Peak no.	RT (min)	Precursor	Mass	Formula	Diff (ppm)	Score (%)	Tentative compound identity
1	5.859	191.0558	192.06	C_7_H_12_O_6_	1.98	95.22	Quinic acid
2	5.909	353.0875	354.09	C_16_H_18_O_9_	0.52	99.39	Chlorogenic acid
3	6.361	299.0769	300.08	C_13_H_16_O_8_	0.65	98.43	Salicylic acid beta-d-glucoside
4	6.963	395.0982	350.10	C_17_H_18_O_8_	0.53	99.65	4-Feruloyl-1,5-quinolactone
5	7.039	137.0243	138.03	C_7_H_6_O_3_	1	99.1	3,4-Dihydroxybenzaldehyde
6	7.139	173.0454	174.05	C_7_H_10_O_5_	1.26	97.56	Shikimic acid
7	7.29	487.2185	428.20	C_21_H_32_O_9_	0.05	99.06	Taraxacolide 1-*O-b*-d-glucopyranoside
8	7.34	367.1033	368.11	C_17_H_20_O_9_	0.32	99.07	3-*O*-Caffeoyl-4-*O*-methylquinic acid
9	7.416	371.0982	372.11	C_16_H_20_O_10_	0.52	99.56	Dihydroferulic acid 4-*O*-glucuronide
10	7.667	693.2772	634.26	C_32_H_42_O_13_	−1.01	98.93	Myricanene A 5-[arabinosyl-(1→6)-glucoside]
11	7.817	477.0678	478.08	C_21_H_18_O_13_	−0.49	99.26	Quercetin 3′-*O*-glucuronide
12	7.842	301.0353	302.04	C_15_H_10_O_7_	0.17	98.58	Hieracin
13	7.842	675.2663	630.27	C_33_H_42_O_12_	−0.48	99.48	13-Acetyl-9-dihydrobaccatin III
14	8.018	505.0982	506.11	C_23_H_22_O_13_	0.98	98.38	Quercetin 7-(6′′-acetylglucoside)
15	8.068	429.2123	384.21	C_20_H_32_O_7_	2.07	96.07	Cinnzeylanol
16	8.081	515.1191	516.13	C_25_H_24_O_12_	0.91	99.11	4,5-Di-*O*-caffeoylquinic acid
17	8.219	461.0719	462.08	C_21_H_18_O_12_	1.14	97.85	Scutellarein 7-glucuronide
18	8.244	923.1505	864.14	C_40_H_32_O_22_	2.07	96.24	2′′,3′′,6′′-Trigalloyliriflophenone 3-C-glucoside
19	8.244	973.5001	974.51	C_48_H_78_O_20_	1.34	98.01	Madecassoside
20	8.244	1019.506	974.51	C_48_H_78_O_20_	1.15	98.39	Asiaticoside B
21	8.269	471.2229	426.23	C_22_H_34_O_8_	1.54	97.12	Cinnzeylanine
22	8.47	515.1189	516.13	C_25_H_24_O_12_	1.12	98.22	4,5-Di-*O*-caffeoylquinic acid
23	8.52	601.1202	602.12	C_28_H_26_O_15_	−0.01	99.06	Eriodictyol 7-(6-galloylglucoside)
24	8.57	557.1293	558.14	C_27_H_26_O_13_	1.31	98.58	Piceatannol 4′-galloylglucoside
25	8.771	1003.51	958.51	C_48_H_78_O_19_	2.39	94.59	Mabiogenin 3-[rhamnosyl-(1→6)-[glucosyl-(1→2)]-glucoside]
26	8.847	395.098	350.10	C_17_H_18_O_8_	1	98.72	4-Feruloyl-1,5-quinolactone
27	9.072	1061.515	1062.52	C_51_H_82_O_23_	2.21	95.26	26-Desglucoavenacoside B
28	9.098	529.1351	530.14	C_26_H_26_O_12_	−0.15	98.67	1-Feruloyl-5-caffeoylquinic acid
29	9.148	563.3433	518.35	C_27_H_50_O_9_	−0.64	75.07	3-*O*-l-Rhamnosyl-3-hydroxydecanoyl-3-hydroxydecanoic acid
30	9.361	1003.511	958.51	C_48_H_78_O_19_	1.7	97.48	Asiaticoside
31	9.374	615.1349	616.14	C_29_H_28_O_15_	1.23	98.77	3,5-Dicaffeoyl-4-succinoylquinic acid
32	9.587	329.0671	330.07	C_17_H_14_O_7_	−1.15	99.29	7,3′,4′-Trihydroxy-3,8-dimethoxyflavone
33	9.888	987.5167	942.52	C_48_H_78_O_18_	0.67	99.38	Soyasapogenol B 3-*O*-[*a*-l-rhamnosyl-(1→4)-*b*-d-galactosyl-(1→4)-*b*-d-glucuronide]
34	10.277	577.1351	578.14	C_30_H_26_O_12_	0.81	93.65	Procyanidin B1

a RT: retention time.

### 3.2 Synthesis of CAPE-AgNPs

For reproducible synthesis of bio-fabricated AgNPs, 5 mg mL^−1^ of CAPE solution was prepared in ethanol : water (1 : 3), 5 mL of the solution was adjusted to pH 9.0 with NaOH, then added to 95 mL of AgNO_3_ solution (0.5 to 4.0 mM) while stirring at room temperature. The multitudinous phenolics can act as a potent reductant and stabilizer for the reduction of AgNO_3_. Research abounds where phytoconstituents in various plants parts have been exploited for biogenic synthesis of AgNPs.^22,35,36^ Biogenic synthesis of nanoparticles follows the general nucleation and growth mechanism. These reactions are kick-started ones the Gibb's free energy changes of the reaction are less than zero.^37^ Moreover, the nucleation and growth processes of nanoparticles synthesis are dependent on several factors such as: pH, temperature, concentration, type of precursor, reducing and stabilizing agent and the molar ratio of precursor to stabilizer.^38^ In the present CAPE mediated synthesis of monodispersed AgNPs, Ag^+^ are reduced in solution by CAPE, by receiving an electron from CAPE, to switch from a positive valence state to a zero valent configuration, followed by nucleation and growth process. These reactions are aptly depicted in the equations below using a notable phenolic present in CAPE (chlorogenic acid, CA) as a decoy of reductive bioactives. At the beginning, chlorogenic acid reduces positive valence state Ag^+^ very slowly, while it is converted to a radical (eqn (1)). This radical reacts with more Ag^+^ in solution rather vigorously, as a result, chlorogenic acids are oxidized to their respective ketone analogues, with concomitant formation of zero valence Ag^0^ (eqn (2)). The formed Ag^0^ are of very small particles diameter and are highly unstable.^39^ Ag^0^ acted as a catalyst towards further reduction of more Ag^+^ in solution to form an intermediate specie Ag_2_^+^ (eqn (3)). These intermediate Ag_2_^+^, further coalesce (eqn (4)), then grow/increase in size to form another intermediate species Ag_4_^2+^ in the popular Ostwald ripening phenomenon.^40^ In the final stage of CAPE synthesis of AgNPs, ripe nanoparticles are further stabilized by other phenolics to prevent particles aggregation through adsorption (eqn (5)).1
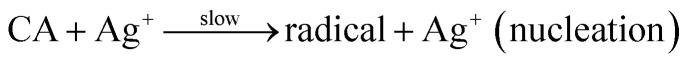
2
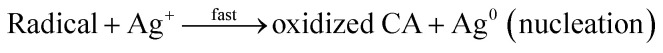
3
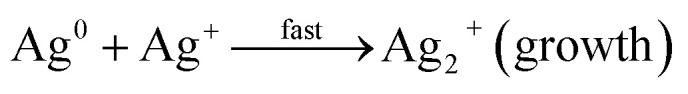

4 Ag_2_^+^ + Ag_2_^+^ → Ag_4_^2+^ (growth)

5
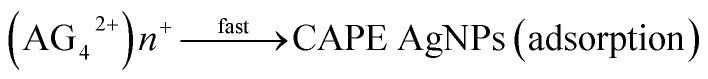


**Fig. 2:**
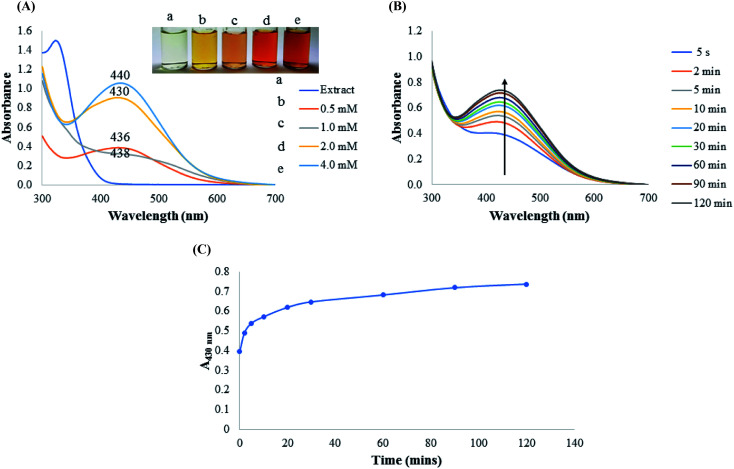
(A) Synthesis of CAPE-AgNPs under different concentrations of AgNO_3_. Inset shows the photographic images of CAPE-AgNPs with (a) CAPE extract (b) 0.5 mM (c) 1.0 mM (d) 2.0 mM and (e) 4.0 mM AgNO_3_ (B) kinetics of CAPE-AgNPs synthesis under 2.0 mM AgNO_3_ within time of 0–120 min and (C) Plot of absorbance at 400 nm of CAPE-AgNPs with time.

As it is generally known, the stability of colloidal metal nanoparticles is dependent on several parameters. From the outset, we investigated the effect of concentration of AgNO_3_ towards AgNPs synthesis, by varying concentration from 0.5 to 4.0 mM. As shown in Fig. 2A, different concentrations of AgNO_3_ resulted in the formation of different AgNPs with distinct visual images (Fig. 2A inset). The absorption spectra collected between 300–700 nm showed maximum absorption at 436 nm (0.5 mM), 438 nm (1.0 mM), 430 nm (2.0 mM), 440 nm (4.0 mM). Optimization condition revealed that the absorbance values of AgNPs synthesized with 2.0 and 4.0 mM AgNO_3_ (Fig. 2A-d and e) are much higher than those synthesized at 0.5 and 1.0 mM AgNO_3_ (Fig. 2A-b and c). It has been speculated that nano-sized AgNPs with maximum absorption spectra between 400–450 nm are of close size distributions, with mostly monodispersed morphology.^18^ It is worth mentioning that the sharp peaks between 430–440 nm of AgNPs result from the electronic oscillation of the conduction electrons on nanoparticles surfaces as a result of interaction with the electromagnetic radiation. This phenomenon is known as the surface plasmon resonance (SPR), which imbue metals nanoparticles with ability to serve in diverse applications and have thus revolutionized the nanotechnology field. Since a red shift in absorption spectra of nanoparticles are often associated with increased particles sizes, CAPE-AgNPs synthesized with 2.0 mM AgNO_3_ having maximum absorption spectrum at 430 nm was used for further investigation. Fig. 2B, shows the kinetics of CAPE-AgNPs synthesis at 2.0 mM AgNO_3_. As can be observed, the absorbance and the intensity at 430 nm increases as a function of time. A plot of absorbance at 430 nm against time (Fig. 2C), shows a swift synthesis kinetics, that is completed at about 90 minutes. Thus, reproducible synthesis of CAPE-AgNPs was achievable at 90 min.

### 3.3 Materials characterization

**Fig. 3:**
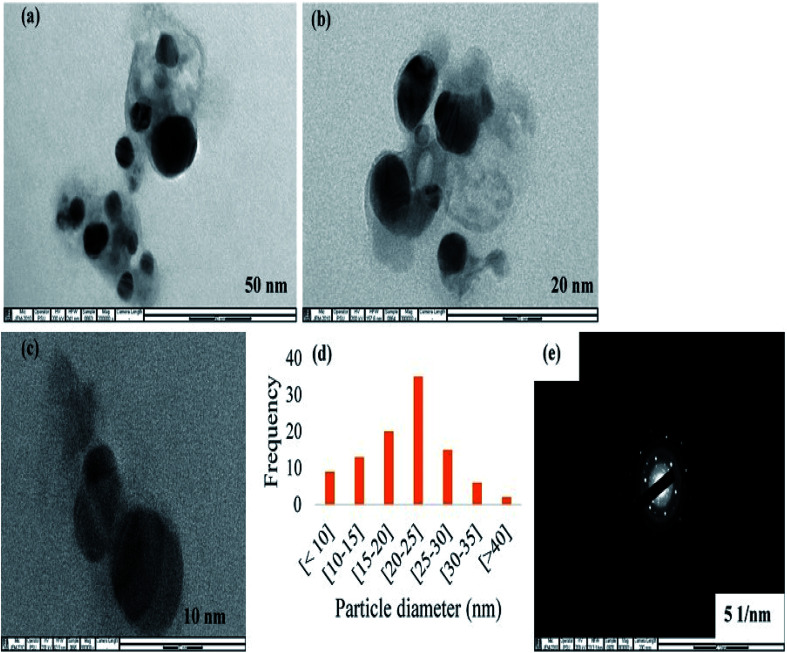
TEM images of CAPE-AgNPs at (a) 50 nm (b) 20 nm (c) 10 nm (d) histogram plot of 100 particles from image J software and (e) selected area electron diffraction images.

CAPE-AgNPs synthesized under our optimized conditions was obtained after three cycles of centrifugation and wash in ultra-pure water. The cleaned CAPE-AgNPs powder obtained through lyophilization was subjected to different instrumental analyses for characterization. Fig. 3 shows the result of the images observed under a transmission electron microscope (TEM). As can be seen, the particles of CAPE-AgNPs are spherical and dispersed. Fig. 3a–c. Fig. 3d, shows the histogram plot of the particles observed with an Image J software. It shows a significant particle distribution around 20–25 nm. The selected area electron diffraction (SAED) image of CAPE-AgNPs is shown in Fig. 3e. The crystalline nature of AgNPs are conspicuously observed as different circles attributed to the different diffraction structure of AgNPs.

**Fig. 4:**
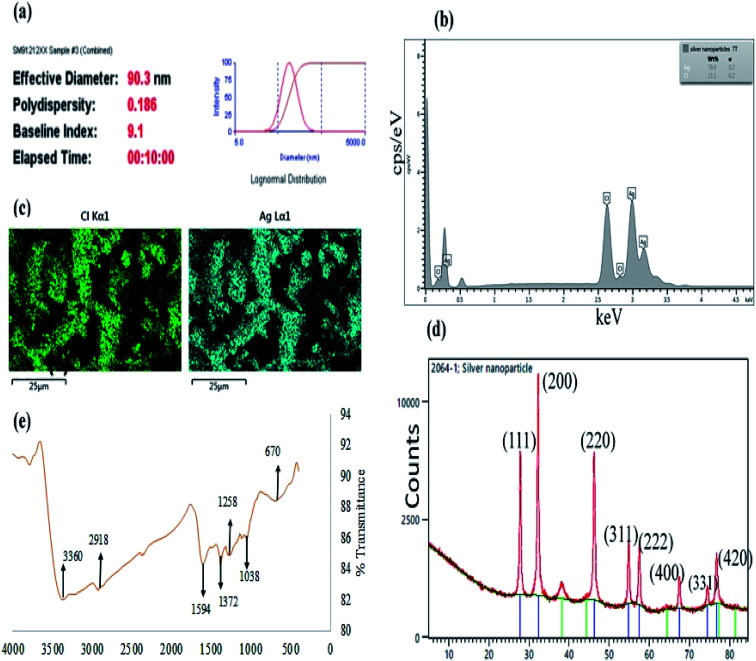
(a) Particles diameter estimate of CAPE-AgNPs from DLS (b) energy dispersive X-ray analysis of CAPE-AgNPs (c) selected elemental mapping of CAPE-AgNPs (d) FTIR Spectrum of CAPE-AgNPs and (e) X-ray diffraction of CAPE-AgNPs.

Moreover, particles diameter of CAPE-AgNPs was also investigated using a dynamic light scattering (DLS) instrument. DLS is one of the most reliable techniques used in verifying colloidal nanoparticles sizes. It should be noted that the particle sizes obtained from DLS are often much larger than the results from TEM. This is because, DLS is used in an aqueous environment and are thus prone to estimating particle sizes in addition to the hydration sphere.^41^Fig. 4a, shows DLS result of CAPE-AgNPs, with average hydrodynamic diameter of 90.3 nm and a poly dispersity index (PDI) of 0.186, attesting to the mono-dispersity of the particles. Colloidal nanoparticles with PDI greater than 0.4 are generally attributed to particles having poly dispersed morphology.^42^ Zeta potential analysis of colloidal metal nanoparticles is another important technique imbued with capacity to reveal information about the stability of nanoparticles. CAPE-AgNPs zeta potential was estimated on a Nano Zetasizer and a value of −28.7 mV was obtained. The negative charges show CAPE-AgNPs are stable in solution and are covered with biomolecules offering stability against facile aggregation. Generally, colloidal nanoparticles with zeta potential values in the range of ±30 mV are assigned to highly stable and dispersed particles.^43^ Thus, CAPE-AgNPs zeta value further validated its stability through phenolics capping on it surfaces.

Furthermore, the elemental compositions of CAPE-AgNPs was investigated by carrying out an energy dispersive X-ray spectroscopic (EDS) analysis. As shown in Fig. 4b, a sharp peak of Ag (78.9%) was observed at 3 keV and another peak of Cl (21.2%) at 2.5 keV. The elemental mapping of CAPE-AgNPs (Fig. 4c), shows the presence of Ag and Cl.

The crystalline nature of CAPE-AgNPs was obtained through X-ray powder diffraction (XRD) analysis. Fig. 4d, reveals major peaks at 27.84, 32.25, 46.20, 54.83, 57.46, 67.45, 74.45, and 77.45 which are located on 2 theta degree of the XRD spectra (111), (200), (220), (311), (222), (400), (331), and (420) crystalline surfaces, which is in conformity with ICDD number (00-031-1238). This result confirmed the face-centered-cubic crystallographic structure of AgNPs. Similar results have been reported by.^44–46^ Moreover, the Scherrer equation, [*D* = *kλ*/*β* cos *θ*] can be used to estimate the particle crystalline sizes of synthesized nanoparticles where *D* is the average crystalline size of nanoparticles, *k* is assigned 0.9 value, *λ* is the wavelength of the X-ray radiation source and *β* is the angular full width at half maximum (FWHM) of the highest peak at (200) diffraction angle *θ*. The estimated average crystalline size is about 16 nm.

In order to probe the presence of bioactives on the surfaces of CAPE-AgNPs, functional group elucidation was investigated with a Fourier transform infrared (FTIR) spectroscopy. Fig. 4e shows the FTIR spectrum of CAPE-AgNPs. Major peaks can be identified at 3360, 2918, 1594, 1372, 1258, 1038, and 670 cm^−1^. The peak at 3360 cm^−1^ is attributed to O–H stretching of OH in phenols and alcohols, peak at 2918 cm^−1^ is due to C–H stretching aliphatic groups, the peak at 1594 cm^−1^ can be attributed to C=C or C=O stretching of amide,^39^ peak at 1372 and 1258 cm^−1^ is assigned to C–C stretching of (in ring) of aromatic compounds^47^ and C–N stretching of amines, the peaks at 1038 cm^−1^ can be assigned to the C–O–C and C–OH stretching vibrations of secondary alcohols. The observed functional groups on CAPE-AgNPs show that CAPE-AgNPs are stabilized by high OH possessing biomolecule probably derived from phenolics, saponins or sugars (Table 2).

### 3.4 Antibacterial activity

Plant phenolic extracts are excellent sources of bioactive antimicrobial compounds. In this study, CAPE showed an antimicrobial activity with MIC and MBC range of 10 to >40 mg mL^−1^ against the tested bacteria isolates. The antimicrobial activities of extracts from the leaves and root of *C. asiatica* have been previously reported with MIC range of 10 to 20 mg mL^−1^ and 5 to >10 mg mL^−1^.^46,48^ As shown in Table 3, the green synthesized AgNPs demonstrated excellent antibacterial activity against the tested isolates with MIC of 0.625 to 1.249 μg mL^−1^ and MBC range of 2.498 to 9.994 μg mL^−1^. The antimicrobial mechanisms of AgNPs are not elaborately understood, however it is suggested that the binding of the positively charge silver ions to the negatively charged bacterial cell wall components facilitates the penetration of the AgNPs across the membrane resulting in disruption of cell membrane, disintegration of DNA and interaction with cellular enzymes, transport proteins and other cytoplasmic components resulting in cell death.^49^ Moreover, the efficacy of some notable organelles such as mitochondria, Golgi apparatus, endoplasmic reticulum is severely compromised, resulting in reduced or interrupted transmembrane electron transport^50^ with devastating consequences.

**Table 3:** Antimicrobial activity of CAPE and CAPE-AgNPs

Bacterial isolates	Extract		CAPE-AgNPs	
	MIC–MBC (mg mL^−1^)		MIC–MBC (μg mL^−1^)	
*B. cereus*	10	40	0.625	2.498
*E. coli*	20	>40	0.625	2.498
*L. monocytogenes*	20	>40	1.249	9.994
*P. aeruginosa*	20	40	0.625	2.498
*S. aureus*	10	>40	1.249	4.997

### 3.5 Catalytic applications of CAPE-AgNPs

#### 3.5.1 Catalytic reduction of 4 NP

**Fig. 5:**
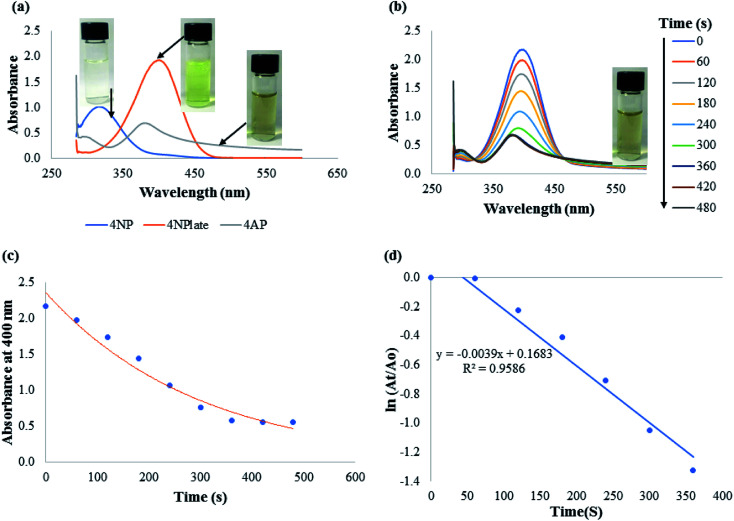
(a) UV-vis absorption plot of 4 NP, 4 NPlate and 4 AP. Inset shows the photographic images (b) UV-vis absorption spectra of 4 NPlate reduction with time. Inset shows the photographic image of the degradation product (c) plot of absorbance at 400 nm with time and (d) linear plot of ln(*A*_t_/*A*_o_) *vs.* time.

Nitrophenols belong to a group of hazardous wastes generated as part of several industrial applications. 4-Nitrophenol is one of the heavily produced nitrophenols. As a matter of fact, 4-NP is listed on the priority hazardous materials list. 4-NP is used to manufacture drugs, insecticides, fungicides, as a pH indicator owing to its colour changing capacity from colourless to an intense yellow in an alkaline medium and for leather darkening.^51^ Inhalation or ingestion of 4-NP in human is marked with deleterious consequences such as drowsiness, nausea, headache *etc.* As a result of its widespread usage, 4-NP can pollute water bodies, hence efforts are consistently geared towards its break down. Green synthesized nanoparticle-mediated catalytic degradation of 4-NP have been well reported.^39,52,53^ We investigated catalytic reduction of 4-NP with CAPE-AgNPs. As shown in Fig. 5a, 4-NP displayed a sharp absorption intensity at 319 nm resulting from the n–π* transition with a faint yellow color. The addition of NaBH_4_ to 4-NP solution resulted in a deep yellow coloration with absorption peak at 400 nm, due to the formation of 4-nitrophenolate ion.^54^ The ability of nanomaterials to mediate electron relay transfer in catalytic degradation of pollutants have been well studied. The high surface area of nanomaterials provide platform for adsorption of both the electron donor or nucleophile (NaBH_4_), to the electron acceptor or electrophile (pollutants), is the most adduced mechanism. As a result, nanomaterials surfaces adsorb in proximity the electron donor and acceptor, consequently lowering the bond dissociation energy of the reaction.^55^ In the absence of CAPE-AgNPs, this solution was stable for over 5 days, attesting to the inability of NaBH_4_ to degrade 4-NP alone. However, the addition of CAPE-AgNPs led to rapid color transition of 4-nitrophenolate to colorless. The absorption spectra reduce in intensity with the generation of another peak at 300 nm. The peak at 300 nm is due to the formation of 4-aminophenol, the reduction product of 4-NP. Fig. 5b shows the changes in the absorption spectra of the mixture of 4-NP, NaBH_4_ and CAPE-AgNPs. The reduction in absorbance at 400 nm was monitored at 1 minute interval. Fig. 5c shows the plot of absorbance at 400 nm against time in seconds, which decreased as time increased. Moreover, the absorbance at 300 nm owing to the formation of 4-AP increases in intensity, which confirms that the concentration of 4-AP increases as a function of time. Since the concentration of the reductant (NaBH_4_), is much higher than other reacting species, the kinetics of 4-NP catalytic reduction are often taken as pseudo-first order. The equation ln(*A*_t_/*A*_o_) = −*kt* suffices in this case, for the estimation of reaction rate constant. *A*_t_ and *A*_o_ are the absorbance monitored at 400 nm of absorbance at any time *t* (*A*_t_) and the initial or starting absorbance value (*A*_o_) respectively, *k* is the rate constant, while *t* is the time. Thus, a plot of ln(*A*_t_/*A*_o_), *vs.* time can be used to estimate the rate constant of the reaction. As shown in Fig. 5d, plot of ln(*A*_t_/*A*_o_) *vs.* time yielded a linear plot with slope of 3.9 × 10^−3^ s^−1^, with *r*^2^ value of 0.959. The slope of the plot represents the *k* value of the catalytic degradation of 4 NP mediated by CAPE-AgNPs. This value was compared with plethora of other nanoparticles reported to degrade 4-NP. As can be observed in Table 4, CAPE-AgNPs showed better capacity, attributable to its high surface area to volume ratio resulting from phenolics capping.

**Table 4:** Comparison of CAPE-AgNPs with reported nanomaterials towards pollutants degradation^a^

Pollutant	Nanomaterials for catalytic degradation	Rate constant (*k*)	References
4 NP	Co-WP-CC	11.1 × 10^−3^ s^−1^	57
	AuNPs@fungus *Trichoderma*	4.9 × 10^−3^ s^−1^	58
	PtNPs@PDDA	0.5 × 10^−3^ s^−1^	59
	Cu–Ag BMNPs	4.05 × 10^−3^ s^−1^	60
	CAPE-AgNPs	3.9 × 10^−3^ s^−1^	This work
MB	AgNPs@*Zanthoxylum armatum*	1.44 × 10^−3^ min^−1^	61
	Fe_3_O_4_@TA/Ag	68.5 × 10^−3^ min^−1^	41
	CAPE-AgNPs	54.7 × 10^−3^ min^−1^	This work
CR	AgNPs@bacterial exopolysaccharide	2.6 × 10^−3^ s^−1^	62
	AuNPs@*Dalbergia-coromandeliana*	4.5 × 10^−3^ s^−1^	63
	CAPE-AgNPs	5.6 × 10^−3^ s^−1^	This work

a PDDA = poly(diallyldimethylammonium chloride), PtNPs = platinum nanoparticles, BMNPS = bimetallic nanoparticles, TA = tannic acid.

#### 3.5.2 Catalytic reduction of MB

**Fig. 6:**
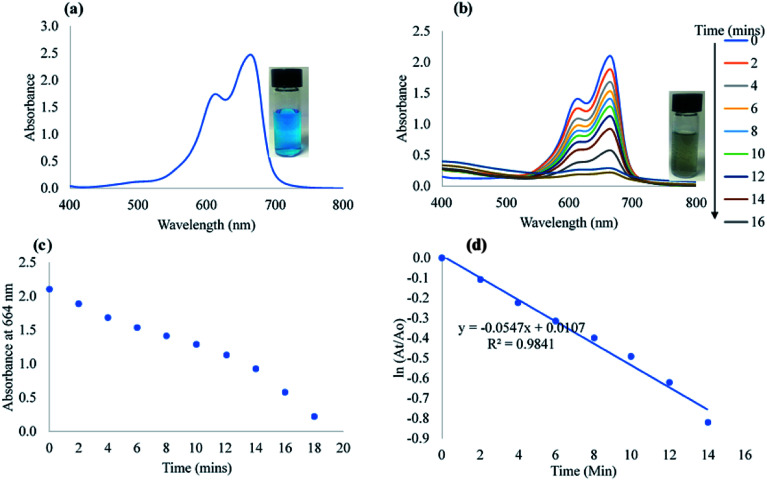
(a) UV-vis absorption plot of MB. Inset shows the photographic image (b) UV-vis absorption spectra of MB reduction with time. Inset shows the photographic image of the degradation product (c) plot of absorbance at 664 nm with time and (d) linear plot of ln(*A*_t_/*A*_o_) *vs.* time.

MB is another common water pollutant. An aromatic heterocyclic dye that is heavily used in the textile industry. MB shows a deep blue coloration with prominent absorption spectra at about 664 nm owing to the n–π* transition and a tail peak at about 612 nm (Fig. 6a) resulting from MB dimerization in solution.^56^ The addition of NaBH_4_ to MB in the absence of CAPE-AgNPs is a slow reduction process that takes much longer time for MB reduction. However, the addition of CAPE-AgNPs to a mixture of NaBH_4_ and MB could be observed to proceed to colorless within sixteen minutes. The color change of MB from blue to colorless signifies the conversion of MB to leucomethylene blue (leucoMB). Fig. 6b shows the reduction in absorbance at 664 nm, which decreased with time. A plot of absorbance at 664 *vs.* time is shown in Fig. 6c, while a linear plot of ln(*A*_t_/*A*_o_) *vs.* time (Fig. 6d), furnished a slope of 54.7 × 10^−3^ min^−1^ with *r*^2^ value of 0.984.

The rate constant of CAPE-AgNPs catalytic degradation of MB 54.7 × 10^−3^ min^−1^, was compared with other reported works. As shown in Table 4, CAPE-AgNPs demonstrated a satisfactory degradation capacity.

#### 3.5.3 Catalytic reduction of CR

**Fig. 7:**
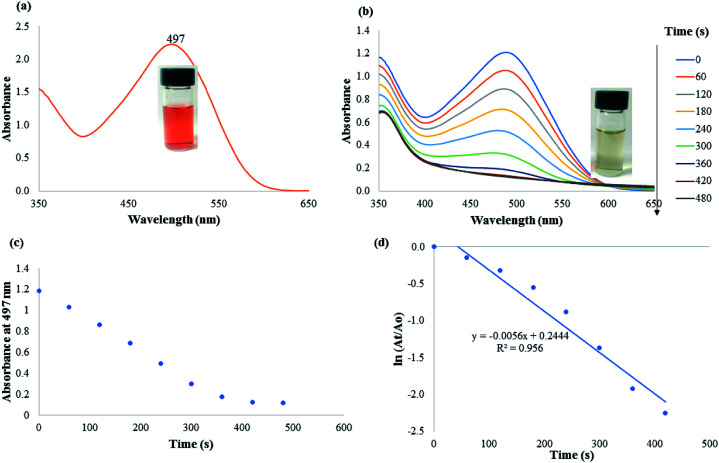
(a) UV-vis absorption plot of CR. Inset shows the photographic image (b) UV-vis absorption spectra of CR reduction with time. Inset shows the photographic image of the degradation product (c) plot of absorbance at 497 nm with time and (d) linear plot of ln(*A*_t_/*A*_o_) *vs.* time.

Congo red (CR), is an anionic azo dye used extensively in the textile and rubber industry. It is toxic to many organisms and has been implicated in carcinogenesis and mutagenesis. It shows a sharp absorption maximum at 497 nm with intensely reddish color (Fig. 7a). The absorption at 497 nm is due to π–π* transition. The addition of CAPE-AgNPs to a mixture of CR and NaBH_4_ resulted in a swift color change from red to colorless, with attendant reduction in absorbance at 497 with time (Fig. 7b). The plot of absorbance at 497 against time is shown in Fig. 7c, while a plot of ln(*A*_t_/*A*_o_) *vs.* time as depicted in Fig. 7d, generated a linear plot with a slope of 54.7 × 10^−3^ min^−1^, with *R*^2^ value of 0.956. The rate constant of CAPE-AgNPs catalytic reduction of CR 54.7 × 10^−3^ min^−1^, was compared with other nanomaterial mediated catalytic reduction, Table 4. The rate constant obtained was comparable to or better than some of the reported works, attesting to the efficacy of CAPE-AgNPs to function in multi-pollutants breakdown.

Moreover, comparison of the reduction efficacy of CAPE-AgNPs towards MB and CR, though of same concentration in the reaction mixture must have stemmed from the differences in their structures. While CR is an anionic azo dye with a diazo group (–N=N–), in their structure, MB on the other hand is a cationic dye, without an azo group. The diazo group is very prone to attack from reducing agents much more than other classes of dyes, hence the significant time differences in their reduction time.

## 4. Conclusion

In conclusion, we have synthesized a spherical and monodispersed biogenic AgNPs using *C. asiatica* phenolic extract (CAPE), tagged CAPE-AgNPs. An UHPLC-ESI-qTOF-MS analysis of the extracted CAPE sample confirmed the presence of copious amounts of different classes of phenolics and other bioactives. The as-synthesized AgNPs was subjected to several instrumental characterizations in order to probe and obtain information such as size, shape or morphology. CAPE-AgNPs of average diameters between 20–25 nm were obtained after optimizing synthesis conditions. CAPE-AgNPs was synthesized within 90 minutes at room temperature, thus avoiding energy consumption. The anti-bacterial activity of CAPE-AgNPs was investigated against some notable foodborne pathogenic bacterial strains. We further exploited the catalytic effectivity of CAPE-AgNPs against some notable environmental pollutants (4 NP, MB and CR). It was found that the catalytic capacity of CAPE-AgNPs was comparable to most of the works reported. This is as a result of the significantly high surface area of CAPE-AgNPs which facilitated facile adsorption of pollutants and the electron donor NaBH_4_ towards catalytic reduction. Thus, this work, offers a potential path towards wastewater treatment involving the transformation of toxic dye pollutants into less harmful components as well as control of pathogenic bacterial strains.

## Conflicts of interest

There are no conflicts to declare.

## Supplementary Material
